# Discovery of a Hidden Schistosomiasis Endemic in the Salamat Region of Chad, Africa

**DOI:** 10.9745/GHSP-D-20-00703

**Published:** 2022-02-28

**Authors:** Timothy Visclosky, Andrew Hashikawa, Eric Kroner

**Affiliations:** a Department of Emergency Medicine, University of Michigan, Ann Arbor, MI, USA.; b The Evangelical Alliance Mission, Grapevine, TX, USA.

## Abstract

There is currently a severe paucity of rural epidemiologic data on urogenital schistosomiasis in the Republic of Chad in north-central Africa that is hindering national control strategies. Our study describes a mobile medical team’s 4-year effort to collect data and provide mass therapeutic and preventive chemotherapy in the Salamat Region of Chad, a previously uninvestigated rural area. To overcome severe limitations in time, resources, support, and infrastructure, the team employed several time- and cost-saving techniques that included: (1) traveling by request and referral to utilize local knowledge to find the areas of highest need, (2) conducting convenience sampling for screening, (3) using simple but validated tools to expedite treatment and data gathering, and (4) working within cultural contexts to find and treat the most school-age children possible. The team managed a total of 11,832 patient encounters and found a 55% rate of hematuria (n=6,495) among school-age children, roughly double previous estimates. Rates of hematuria were higher in males (61%, n=3,955/6,466) than females (48%, n=2,301/4,806) and among adolescents (> 65% in all age groups aged 10 years and older). These methods outline an efficient and effective strategy implemented under real-world conditions to enact therapeutic and preventive chemotherapy campaigns in resource-limited settings by engaging village leadership and developing locally driven solutions. Our data highlight the need for continued local epidemiologic efforts to treat the substantial number of children severely affected with schistosomiasis.

## Abstract (precis)

A mobile medical team used numerous time- and cost-saving techniques to provide therapeutic and preventive chemotherapy to nearly 12,000 patients while uncovering a hidden urogenital schistosomiasis endemic in the Salamat Region of Chad, Africa.

## Key Messages

- There is a 55% prevalence of urogenital schistosomiasis among school-age children in the Salamat Region of Chad, Africa.
- These findings provide vital epidemiologic data and outline an efficient, culturally sensitive, and reproducible method for conducting mass chemotherapy campaigns in resource-limited settings.
- National stakeholders should continue to evaluate the local epidemiology of schistosomiasis and use that information to target preventive chemotherapy.
- Program managers can improve efficiency while maintaining efficacy by following requests and referrals to find areas of highest need, using convenience sampling for screening, employing simple but validated tools for treatment and data gathering, and working within cultural contexts to maximize impact.

Résumé en français à la fin de l’article.

## INTRODUCTION

Schistosomiasis is classified as a neglected tropical disease by the World Health Organization (WHO) and affects an estimated 240 million people worldwide.^1^^,^^2^ One manifestation of this disease, urogenital schistosomiasis, is caused by the trematode *Schistosoma haematobium* and is propagated by snails in freshwater. Their larvae penetrate human skin and ultimately deposit eggs in various sites within the human host.^2^ Snails’ predilection for stagnant water causes hotspots to arise in many rural areas with limited resources for reporting, testing, or treatment.^3^

Urogenital schistosomiasis is considered a disease of poverty and predominantly affects school-age children (SAC).^4^^,^^5^ Infections are often characterized by hematuria, dysuria, urinary frequency, pelvic pain, and a serum sickness-like illness characterized by ill appearance, rash, fever, and arthralgias.^4^ Infections, if left untreated in children, can cause long-term sequelae such as anemia, growth retardation, cognitive impairment, infertility, and bladder cancer.^4^^,^^5^

These long-term sequelae pose significant public health burdens on endemic regions. The physical and cognitive impacts of these infections continue to affect SAC into adulthood, resulting in ongoing morbidity. The subsequent health, financial, and social burdens affect entire communities.^6^ Though it is difficult to fully quantify, it is currently estimated that sub-Saharan Africa will lose 2.3 million disability-adjusted life-years and US$3.5 billion of economic productivity every year as a direct result of schistosomiasis and soil-transmitted helminthiasis.^7^ Clearly, investments in combating this disease can have far-reaching implications on the endemic communities most affected.

The treatment of this disease, a single dose of praziquantel, is safe, easily administered (using the WHO height-based dosing pole), inexpensive, and effective.^8^ In addition to treating individual cases, praziquantel also serves to interrupt transmission.^9^^,^^10^ Targeted preventive chemotherapy (PC) campaigns are shown to be a cost-effective method for decreasing community spread of urogenital schistosomiasis in resource-limited regions but require accurate and updated local epidemiologic data and working knowledge of local conditions.^9^^,^^10^

The severe lack of epidemiologic data continues because urogenital schistosomiasis has significant regional variation, even within endemic countries.^3^ In 2018, the WHO highlighted how strategic PC campaigns continue to be hampered by incomplete local epidemiologic data, with only 41.6% of the global population requiring coverage actually receiving PC.^11^

The severe lack of epidemiologic data continues because urogenital schistosomiasis has significant regional variation, even within endemic countries.

Recently, the WHO has set a target date of 2030 for eliminating schistosomiasis as a public health problem in endemic countries.^12^ Though recent studies have reaffirmed that this goal is achievable, there remains no consensus on the best approach to community-wide treatment.^9^^,^^10^ For example, many previous reports have suggested using school-based distribution programs to target SAC, but this approach does not accommodate countries such as Chad where many communities have limited school facilities or attendance.^8^ The challenge in schistosomiasis control lies in coordinating the effective and targeted distribution of praziquantel based on local epidemiologic data.

### Regional Context

The Republic of Chad, located in north-central Africa, is an endemic area for schistosomiasis, with over 2.6 million SAC requiring preventive chemotherapy annually.^1^ Like other low-resource countries, the Republic of Chad struggles to control the urogenital schistosomiasis endemic. Treatment remains far below the WHO PC treatment goal of 75% coverage, and the estimated infection rate remains greater than 25%.^1^^,^^11^

Unfortunately, this is unsurprising given the challenges in Chad. The United Nations ranks Chad 187 of 189 countries by the human development index score in 2020.^13^ Contributing to this ranking, Chad notably struggles with both school and health infrastructures.^14^ Over 1.7 million SAC were estimated to be out of school in 2019, making these sites impractical distribution centers for PC campaigns.^15^ Furthermore, there remains only 1 doctor per 28,466 people within the country, resulting in a health system that already struggles to meet the other known endemic diseases, including malaria, HIV, and TB.^14^

**FIGURE 1:**
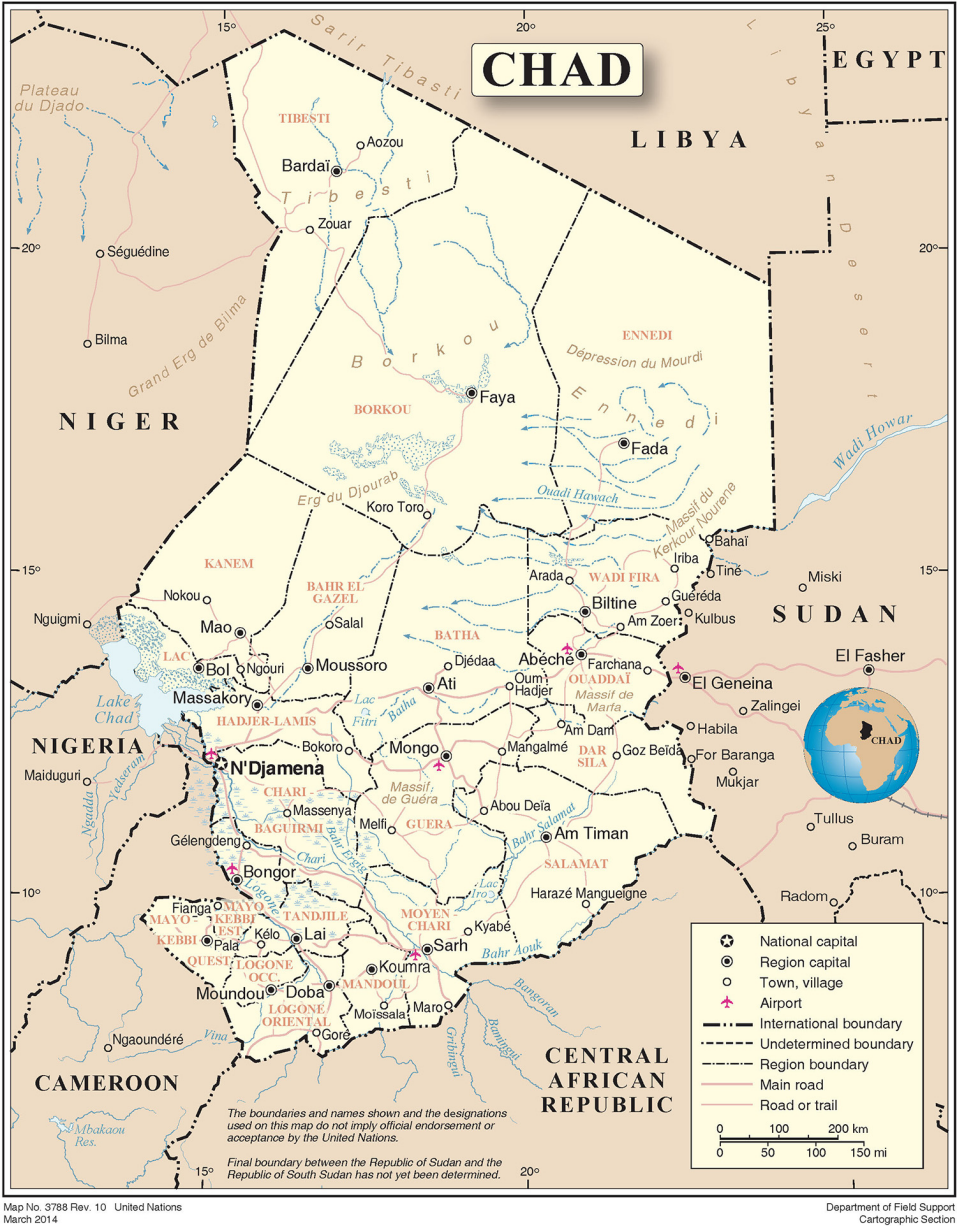
Map of Chad Showing the Salamat Region Source: United Nations Department of Field Support, 2014.

Within Chad, the Salamat Region (the location of our study) is perhaps the least developed (Figure 1). It was designated the “poorest region” in the world by the 2015 Oxford Poverty and Human Development Initiative, with 98% of the population living below the poverty line.^16^ Salamat’s population relies upon seasonal stagnant surface water with sparse sanitation systems, increasing the risk of urogenital schistosomiasis and contributing to the epidemiologic heterogeneity and the continued knowledge gaps regarding focal endemicity.^14^ To our knowledge, only a few studies have previously investigated urogenital schistosomiasis in Chad with no inclusion of the eastern regions.^17^^–^^19^ These studies have primarily reported the prevalence of school- and hospital-based treatment campaigns, excluding large swaths of the population without regular access to these resources.

These real-world conditions in Chad, including limited resources, information, and infrastructure alter how traditional schistosomiasis chemotherapy campaigns can be appropriately implemented. This gap between conventional practice and reality will continue to hinder the already strained national control efforts until it is addressed using alternative and creative strategies.

### Project Development

This study arose after a physician living in the Salamat Region discovered an index case of urogenital schistosomiasis. In August 2015, when the physician treated a village chief’s daughter for a presumed urinary tract infection, it became clear that hematuria was commonplace among the community’s children. The team quickly found that more than half of the village children had gross hematuria. A subset of these urine samples was examined, with 100% having haematobium eggs on microscopy. Further investigation revealed a previously unknown, high-intensity regional endemic. The medical team gradually developed and fine-tuned a methodology for treatment and data collection driven by limited resources and a sense of urgency to treat the greatest number of SAC. The strategy underwent iterative improvements and while there remain areas for growth, the methodology below outlines our experience designing, implementing, managing, and supporting health programs in the real-world conditions of a low-income country.

## METHODS

### Follow the Tip

Though numerous systematic screening methods were considered, significant constraints on time, supplies, and personnel forced the prioritization of high-risk children for treatment. The resultant methodology leveraged local resources to identify the areas of highest need. While this may not be the most systematically rigorous methodology, it bypassed many of the inefficiencies and challenges associated with more systematic approaches in areas with extreme resource limitations.

Village engagement arose through request or referral and relied on local knowledge from health and village authorities. The information received from these groups generated a growing list of “tip-villages,” areas suggested to the team as having high rates of hematuria. Village chiefs, with their intimate knowledge of the community, were perhaps the most invaluable resource. After news spread of our team’s goals, many village chiefs requested a screening visit if they knew of significant hematuria among SAC. As this study continued, a growing network of friends also developed throughout the region. These included local villagers who would reach out if hematuria was known to be prevalent. Health authorities included zone medical personnel and international health workers. Zone medical personnel were kept apprised of our efforts and provided valuable local knowledge and a sustained nexus between the team and community but were often unable to contribute time or material resources. The endemic was signaled, and an appeal was made to national and international health authorities, resulting in a small but significant quantity of praziquantel. Throughout the process, village authorities were prioritized for their updated knowledge of the situation, their desire to collaborate, and their consistently higher response rate.

Village engagement relied on local knowledge, which generated a growing list of “tip-villages,” areas suggested to the team as having high rates of hematuria.

These resources combined to create our path between tip-villages. From 2015 to 2019, the study team traveled from tip-village to tip-village with no shortage of leads. The results, described below, demonstrate that this method effectively targeted the highest-risk groups, as the rate of infection uncovered was nearly double that of the WHO criteria for intervention.^8^

The risk associated with this screening methodology was missing endemic villages. However, the resource constraints involved made more comprehensive screening impossible in this phase of our strategy. Many recommend the use of schools for screening. However, the poor development status of the Salamat Region and their dependence upon subsistence farming (forcing SAC into fields rather than schools) made this strategy unfeasible. We hope to leverage the information gained by our “follow-the-tip” approach to garner more attention, local support, and resources to provide for a more comprehensive approach that is needed.

### Screening

The mobile medical team was organized to run with 1 physician and 2 medical assistants. On the initial visit, the physician would begin by approaching the village chief. After an explanation of urogenital schistosomiasis, the medical team would offer to screen a sampling of children. If the village chief consented, a convenience sampling of children was performed. Children were included if they were aged between 5 and 15 years. They were excluded if they were pregnant or ill-appearing and required immediate referral to the regional health center. The number of children was variable depending upon availability and the size of the village, ranging from 5–30. Urine samples were evaluated for hematuria and were deemed positive if gross hematuria was present on visual inspection or urine dipstick. This method of using macroscopic hematuria and urinary reagent strips has been validated as a method of identifying Schistosoma haematobium infections.^20^ While it is unable to quantify the level of infection, it is a quick and reliable method that has proven utility in low-resource settings.^20^^–^^22^ If 30% or more of the samples were positive for hematuria, villages qualified for universal treatment of schistosomiasis in SAC.^8^ This was considered the minimum criteria for intervention.

**Figure:**
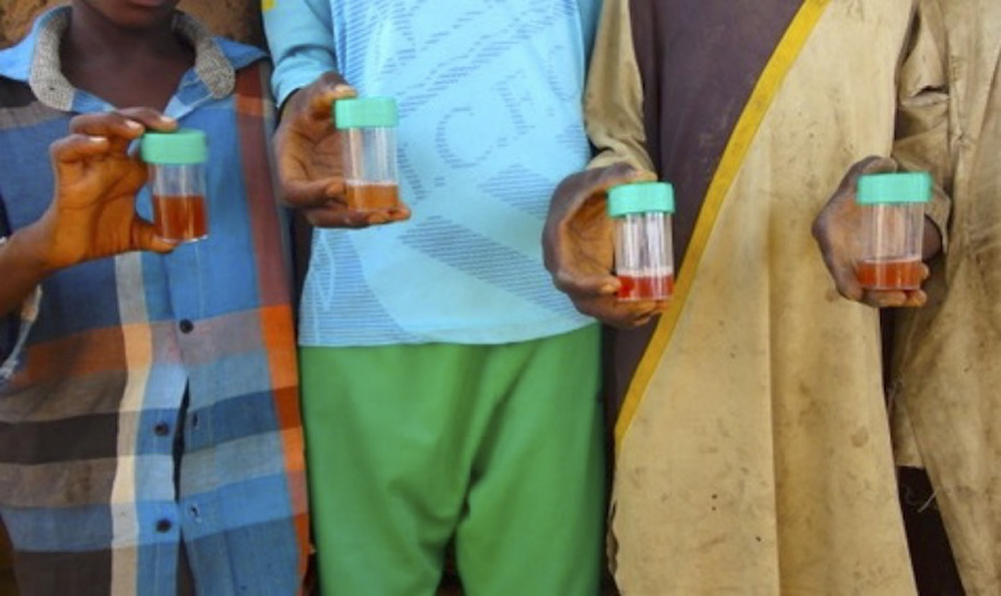
Example of convenience sampling of village children. © 2016 Eric Kroner

### Phase 1: Treatment

The primary goal during Phase 1 of our study was to treat as many highly infected, first-time referral villages as possible given the time and resource constraints. There was no specific target for the number of villages to treat in this phase. The medical team approached, screened, and treated the maximum number of villages given these considerations.

A future date was set for treatment, at which time the medical team returned and the village children were congregated as directed by their caregivers. Children were considered eligible for treatment if they were between the ages of 5 and 15 years. Children were ineligible if they were unable to swallow pills, pregnant or lactating, or ill-appearing and required referral to the regional health center. Eligible patients were given praziquantel pills, dispensed based on their height measured against the WHO tablet pole. As praziquantel was being dispensed, basic information was collected and recorded via paper documentation on all SAC presenting for treatment. The data collected included age, height, and weight, as well as if they saw red-colored urine.

**Figure:**
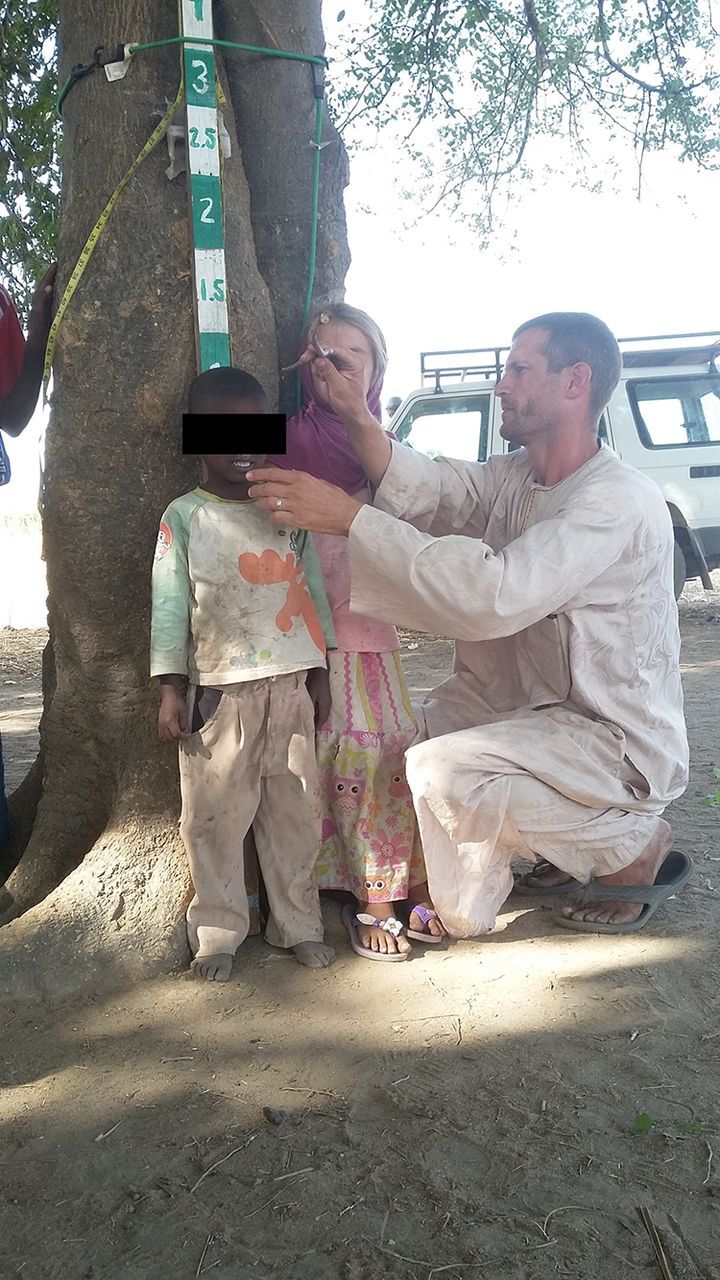
World Health Organization tablet pole being used to measure the height of a child. © 2017 Eric Kroner

### Phase 2: Follow-up

It is understood that the reliance on subsistence farming and poor health care infrastructure nearly guarantees re-exposures among SAC and at least annual treatment visits will ultimately be required. We consider this an achievable goal and will be a top priority in the impending phase 2 of our treatment strategy.

### Findings

During the study period, 82% of villages (n=56/68) screened positive based on WHO standards and qualified for mass treatment with praziquantel. Of those 56 treated villages, 11% (n=6) were visited a second time in a subsequent calendar year. Due to limited time and resources of the medical team, villages that had not yet received any treatment were prioritized over villages requesting a second visit. The total study population was 11,832, with 99.5% (n=11,771) being children. Of the total study population, 91% (n=10,780) were eligible and received treatment with praziquantel; 55% (n=6,646) were male; 41% (n=4,806) were female; and 4% (n=560) were not recorded. The mean age was 7.9 years (standard deviation 4.4). Overall, 55% of patients endorsed gross hematuria (n=6,495), 34% denied hematuria (n=4,041), and 11% were unsure (n=1,296). Of the 56 treated villages, 33 (59%) had a prevalence equal to or greater than 50%.

**FIGURE 2:**
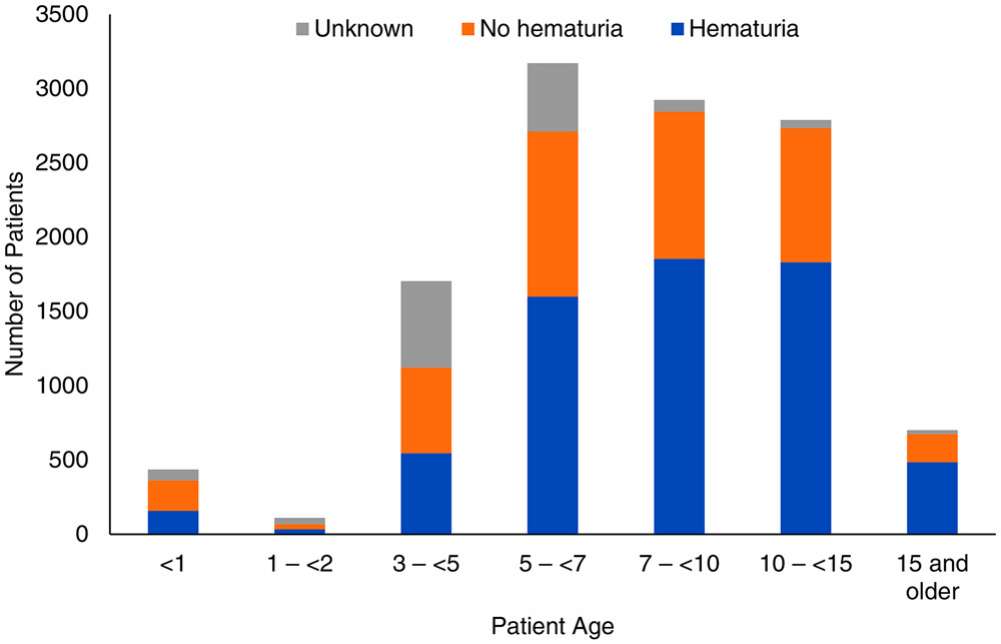
Rates of Hematuria Among School-Age Children Screened for Urogenital Schistosomiasis, by Age, Salamat Region, Chad

**FIGURE 3:**
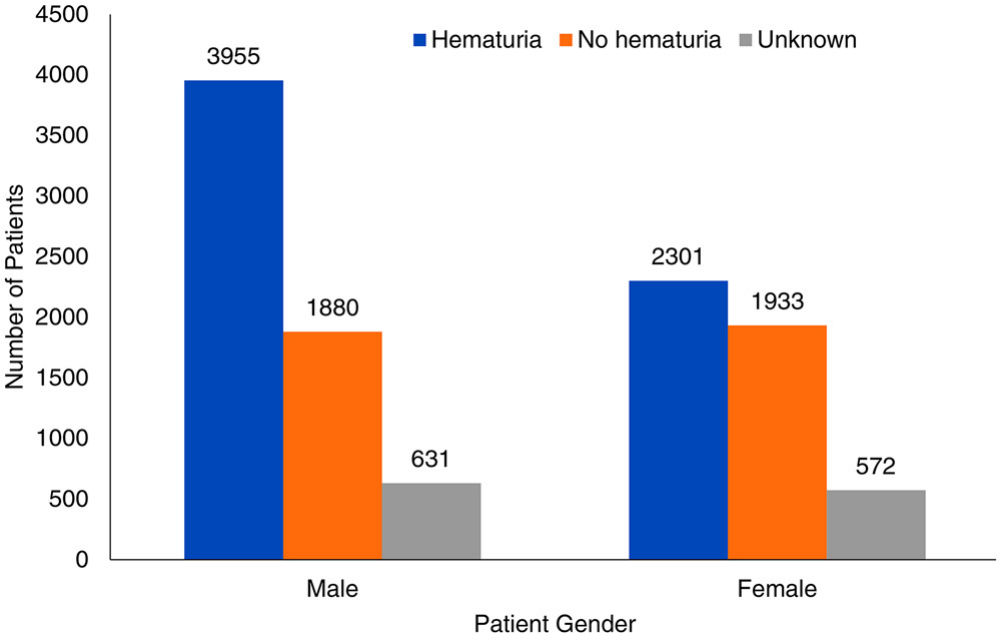
Rates of Hematuria Among School-Age Children Screened for Urogenital Schistosomiasis, by Gender, Salamat Region, Chad

Reported hematuria also increased with age in our study population. The highest prevalence of hematuria was noted in patients aged 15 years and older (69%, n=484/702), followed by children aged 10–15 years (63%, n=1,855/2,923) (Figure 2). Among all age groups, the prevalence was higher in males, with 61% (n=3,955/6,466) reporting hematuria compared to 48% (n=2,301/4,806) for females (Figure 3).

## LESSONS LEARNED

### Utility

While our methodology had limitations, our strategic intervention grew organically from the local realities within Chad and had high utility and advantages over traditional approaches to testing and treatment in locations with extreme resource limitations and high-intensity infection. The use of local knowledge and convenience sampling allows for the rapid identification of high-risk populations and facilitates the first dosing for many who may never have previously received treatment. Our treatment efforts may halt disease progression (even if only for 1 or 2 years) and prevent severe complications. Our approach can be accomplished with limited financial support and can facilitate data collection and the development of a more comprehensive screening strategy.

The use of local knowledge and convenience sampling allows for the rapid identification of high-risk populations and facilitates the first dosing for many who may never have previously received treatment.

**FIGURE 4:**
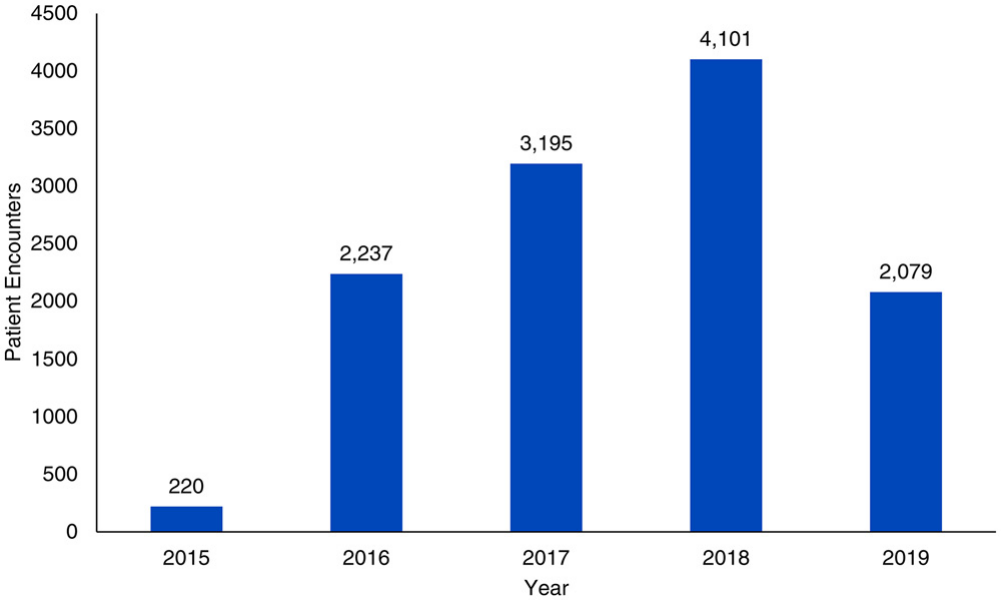
Number of Patient Encounters With a Mobile Medical Team Screening for Urogenital Schistosomiasis, by Year, Salamat Region, Chad

Ultimately, this mobile medical team was plagued by many of the same constraints that routinely affect teams treating endemic urogenital schistosomiasis within extreme resource-limited settings. The development and use of a small mobile team, traveling by request or referral, and using convenience sampling and other validated but straightforward tools increased efficiency, conducting nearly 12,000 patient encounters under the guidance of 1 physician (Figure 4).

### Successes in Sustainability

The primary benefit of this methodology lies in the numerous steps toward sustainability. Engaging the community in both screening and treatment built trust and carved inroads to future collaborations, as demonstrated in the gradual growth of our referral network. After visualizing the actual rates of hematuria in SAC, villages gained knowledge of the disease and increased collaboration with the medical team. Our use of local personnel in the medical team also allowed for a system of education and training for study team members with a permanent presence in the region. Furthermore, this proved to be an efficient method of identifying high-prevalence areas as 82% of the villages screened positive on the initial visit.

The use of convenience sampling of children for screening was born out of necessity but ultimately increased efficiency without sacrificing accuracy.

The tools used in this intervention are also easily attained and recreated. The use of convenience sampling of children for screening was born out of necessity but ultimately increased efficiency without sacrificing accuracy. Despite initial concern that this approach could lead to a misrepresentation of disease burden, there was no evidence that this occurred. All villages that screened positive had high rates of self-reported hematuria when assessed on the return treatment date. While this was a strength in our particular study, it should be noted that areas with different disease prevalence may be more vulnerable to the failings of convenience sampling.

The use of the WHO tablet pole for medication dosing and self-reported hematuria as a marker for urogenital schistosomiasis infection were both evidence-based, time- and cost-saving techniques. Designed for height-based dosing, previous studies have raised concerns that the WHO tablet pole’s validity could be affected by patients’ body habitus and age.^23^ Our treatment population was mostly underweight children, with the majority of patients having a BMI less than 15. In this population, the WHO height-based dosing pole performed well, with more than 98% receiving an appropriate praziquantel dose. The use of self-reported hematuria as a marker of infection, while not the gold standard, is a similarly validated and efficient method for data collection.^20^^–^^22^ These 2 simple but effective tools increased efficiency and allowed for rapid and accurate treatment and data collection under the guidance of only 1 physician.

### Limitations in Sustainability

With the advantage of hindsight, our team would like to place a higher priority on training local nonmedical personnel. This could have been achieved by rotating the nonmedical personnel through positions in the clinic to create redundancy in skillsets and expertise. This would reduce the role of the physician on treatment day and generate increased ownership for local workers. As this study grew out of a sense of urgency, this focus developed near the end of phase 1, later than would be ideal.

The involvement of local medical collaborators and health volunteers was also limited. Repeated attempts to engage local health officials were largely unsuccessful, resulting in a reliance on local, nonmedical personnel. After collecting the data outlined above, we hope to have more success with recruiting both zone medical personnel and community health volunteers in phase 2 as the problem is now well-defined and local villages are already engaged. We will attempt to identify a community health volunteer in each village (or cluster of villages) to be trained in disease recognition and reporting to create a screening network.

The attainment of praziquantel similarly remains a limiting factor. After significant effort, only small donations were obtained from international and governmental health organizations. Moving forward, we hope to leverage the epidemiologic data described above to demonstrate the devastating endemic and solicit more contributions.

There is also a theoretical concern that cultural practices and beliefs may impair the long-term success of our proposed “follow-the-tip” method. In some areas of schistosomiasis endemicity, hematuria is considered a normal and common occurrence and has even been referred to as “male menstruation.”^24^ There can also be reticence to discuss bowel or bladder functions. Because our method relied on villages to self-report and request visits, these beliefs have the potential to interfere with a team’s ability to find highly infected villages. During our study, the team encountered some mild reticence to discuss bodily functions but none that impaired the team’s progression through tips. There was also no obvious stigma assigned to schistosomiasis infections in any of the villages. The ability to explore how these cultural beliefs can potentially impact the “follow-the-tip” method will ultimately require a larger-scale investigation and is recommended as an area of further investigation.

## NEXT STEPS

Phase 2 of this project will focus on building local collaborations, developing a sustainable and comprehensive screening program, and education. As mentioned, this project was initially hampered by the limited capacity of the local health system. However, data collected in phase 1 now demonstrate a previously undocumented urogenital schistosomiasis endemic that has remained hidden and insidious. We hope these data will sound the alarm about the severity of the problem and call other partners alongside the local health department.

Our reliance on “tip-villages” was both necessary and effective for phase 1 but will be refined to achieve a more sustainable comprehensive screening program in phase 2. “Tip-villages” allowed for the rapid identification and treatment of thousands of high-risk individuals. However, it leaves the team at risk of missing some highly infected areas. After defining the problem and garnering more support for our efforts, we hope to move to a more systematic screening approach.

After defining the problem and garnering more support for our efforts, we hope to move to a more systematic screening approach.

Finally, education will be more heavily incorporated into phase 2. In collaboration with local artists, laypeople, and medical professionals, the study team has created a contextualized story flip chart to explain the transmission and treatment of schistosomiasis to address some of the stigmas surrounding the disease. This will be shared with villages on treatment days and will build upon the education already initiated in phase 1 when the extent of the problem was revealed to village authorities. Our educational approach will be the first step in a larger effort to leverage local resources to educate children/villages on how to prevent exposure, identify symptoms, and seek help.

## CONCLUSIONS

Urogenital schistosomiasis is a highly treatable disease with significant public health burdens if unrecognized or untreated. The challenges to management lie in its high degree of regional variation and insidious course. Recently, there was a worldwide call for increased local epidemiologic data in schistosomiasis endemic countries.^9^ If these data can be collected and the WHO goals for disease control can be achieved, recent estimates suggest a greater than US$17 billion in economic benefits and significant school attendance and educational gains would be seen.^25^

Unfortunately, this is a significant burden to place on the many afflicted areas that are resource-limited and focused on other ongoing public health threats. The Salamat Region of Chad is a prime example, with their self-reported PC campaign remaining far below the goals set by the WHO.^1^^,^^11^ By using convenience samples and local referral networks as indicators of high-risk communities, we uncovered a 55% prevalence among SAC in the Salamat Region of Chad, far exceeding the 25% prevalence rate described in other areas of the country.^17^^,^^18^ In this context, our study suggests that the substantial regional penetration variation continues to evade national control efforts. Clearly, alternative strategies to the school-based preventive chemotherapy approach are necessary and must accommodate local conditions.

The mobile medical team faced numerous resource and logistical challenges similar to those found in many resource-limited settings. Our team’s tailored strategy using multiple time- and cost-saving measures, such as traveling by request and referral, implementing convenience sampling, not relying on schools, and employing the validated tools of the WHO tablet pole and self-reported hematuria provided adaptable, efficient, and effective treatment for SAC. Our approach can be a model for other medical teams seeking to not only provide mass PC in low-resource areas but also to collect vital local epidemiologic data to uncover the true disease burden needed to eliminate urogenital schistosomiasis.

## Translation

**En Français**

**Découverte d'une Schistosomiase Endémique Cachée dans la Région de Salamat au Tchad, Afrique**

**Message Clé**

Pendant quatre ans, une équipe médicale mobile composée d'un médecin et de deux assistants médicaux a utilisé de nombreuses techniques permettant d'économiser du temps et de l'argent pour fournir une chimiothérapie thérapeutique et préventive à près de 12 000 patients tout en découvrant une schistosomiase urogénitale endémique cachée dans la région de Salamat au Tchad, en Afrique. Ces résultats fournissent des données épidémiologiques essentielles et décrivent une méthode efficace, culturellement sensible et reproductible pour mener des campagnes de chimiothérapie de masse dans des environnements aux ressources limitées.

**Résumé**

Il y a actuellement une grave pénurie de données épidémiologiques rurales sur la schistosomiase urogénitale dans la République du Tchad, en Afrique centrale du Nord, qui entrave les stratégies nationales de contrôle. Notre étude décrit les efforts déployés pendant quatre ans par une équipe médicale mobile pour collecter des données et fournir une chimiothérapie thérapeutique et préventive de masse dans la région de Salamat au Tchad, une zone rurale qui n'avait jamais été étudiée auparavant. Pour surmonter les graves limitations de temps, de ressources, de soutien et d'infrastructure, l'équipe a utilisé plusieurs techniques permettant d'économiser du temps et de l'argent: 1) se déplaçant sur demande et par référence afin d'utiliser les connaissances locales pour trouver les zones où les besoins sont les plus importants, 2) effectuant un échantillonnage de convenance pour le dépistage, 3) utilisant des outils simples mais validés pour accélérer le traitement et la collecte de données, et 4) travaillant dans des contextes culturels pour trouver et traiter le plus d'enfants d'âge scolaire possible.

L'équipe a géré un total de 11 832 rencontres avec des patients et a constaté un taux d'hématurie de 55% (n = 6495) chez les enfants d'âge scolaire, soit environ le double des estimations précédentes. Les taux d'hématurie étaient plus élevés chez les garçons (61%, n = 3955/6466) que chez les filles (48%, n = 2301/4806) et chez les adolescents (> 65% dans tous les groupes d'âge de plus de dix ans). Ces méthodes décrivent une stratégie efficace et efficiente mise en œuvre dans des conditions réelles pour mener des campagnes de chimiothérapie thérapeutique et préventive dans des contextes où les ressources sont limitées, en faisant appel aux dirigeants des villages et en élaborant des solutions locales. Nos données soulignent la nécessité de poursuivre les efforts épidémiologiques locaux pour traiter le nombre substantiel d'enfants gravement touchés par la schistosomiase.

**Peer Reviewed**

**First published online:** February 18, 2022.
